# Perceived Capability, Social Belonging, and Behavioral Adherence in Adult Group Fitness Classes: A Systematic Review of Psychosocial Predictors

**DOI:** 10.3390/bs16060882

**Published:** 2026-06-01

**Authors:** Xiaoxue Gao, Yong-Gwan Song, Yu Shu, Ana Filipa Silva, Gianpiero Greco

**Affiliations:** 1 Graduate School of Physical Education, Pukyong National University, Busan 48513, Republic of Korea; 19983992687@163.com (X.G.); ygsong@pknu.ac.kr (Y.-G.S.); 2 School of Physical Education and Health, Sichuan Technology and Business University, Meishan 620000, China; 3 Faculty of Sport Sciences and Physical Education, University of Coimbra, 3040-256 Coimbra, Portugal; 4 Department of Translational Biomedicine and Neuroscience (DiBraiN), University of Study of Bari, 70124 Bari, Italy; gianpiero.greco@uniba.it

**Keywords:** group fitness, exercise adherence, self-efficacy, social support, group behavior

## Abstract

Objectives: This systematic review identifies and synthesizes quantitative evidence regarding whether psychosocial constructs, specifically groupness, social support, self-efficacy, enjoyment, and instructor behavior, are associated with behavioral adherence to adult group fitness classes. Methods: Electronic searches were conducted in PubMed, Scopus, and Web of Science. Eligible studies included quantitative analyses of adults in instructor-led group exercise. Risk of bias was appraised using ROBINS-I, and JBI critical appraisal tools, as appropriate to study design. Results: Twenty-one studies met inclusion criteria. Self-efficacy was the most consistently supported domain, with several behavior-specific efficacy measures showing small-to-moderate positive associations with attendance-based adherence, although null findings were also observed when measures were less temporally aligned or when outcomes shifted to post-program continuation. Social-relational and instructor-related constructs showed more context-dependent evidence: social attraction, social cohesion, reliable alliance, group identification, and selected leader behaviors were more consistently favorable than generic social support or broad cohesion measures. Enjoyment and affective response showed the weakest independent evidence, with positive associations mainly when affective constructs were measured close to the attendance decision. Adherence in instructor-led group exercise settings appears to be influenced by perceived capability, social-relational processes, instructor-shaped motivational climate, and affective valuation, although the available evidence is heterogeneous and predominantly observational. Future research should use multilevel longitudinal designs, instructor- and class-level clustering, repeated affective assessment, and causal pathway modeling to test how these mechanisms support repeated attendance and longer-term maintenance.

## 1. Introduction

Adherence to exercise is a relevant issue because consistent participation is required for exercise programs to generate and maintain benefit, and higher adherence has been associated with better outcomes in community-based exercise interventions (Belza et al., 2002; Hicks et al., 2012). Group contexts such as fitness classes are common forms of physical activity, and regular participation in group fitness classes has been associated with lower perceived stress and better quality-of-life indicators in adults (Graupensperger et al., 2019; Yorks et al., 2017). In studies of group-based exercise, adherence has commonly been considered using behavioral indicators such as class attendance, retention or completion, persistence, and dropout (Farrance et al., 2016; Hawley-Hague et al., 2014). Because attendance in instructor-led group fitness classes requires repeated decisions to return to a shared class environment, adherence in this setting can be conceptualized as a behavior emerging from the interaction of individual capability, social opportunity, and motivational processes rather than as a function of exercise exposure alone (Michie et al., 2011).

Several psychosocial constructs are plausible determinants of continued class participation because they correspond to complementary and interacting mechanisms within behavioral-science and exercise-motivation frameworks. Within COM-B, behavior is generated through capability, opportunity, and motivation; applied to instructor-led group fitness, self-efficacy can be interpreted as perceived capability, groupness/cohesion and social support/relatedness as social opportunity, instructor behavior as a social-contextual input, and enjoyment or affective response as affective motivation (McAuley & Blissmer, 2000; Michie et al., 2011; Rhodes & Kates, 2015; Stevens et al., 2018; Teixeira et al., 2012). This mapping is not intended as a static taxonomy. Rather, it frames adherence as a repeated attendance process in which class experiences may feed forward into later confidence, perceived belonging, affective valuation, intention formation, and either maintenance or disengagement (Rhodes & Sui, 2021).

Empirical findings in group exercise are consistent with this integrated account, although the evidence remains uneven across pathways. Intensive longitudinal and observational studies suggest that groupness, social support, competence, relatedness, self-efficacy, affective response, and instructor-related processes may each be associated with class experience, attendance, or persistence in some settings (Golaszewski et al., 2022; Graupensperger et al., 2019; Middelkamp et al., 2016, 2017; Vlachopoulos & Neikou, 2007). However, these findings should be interpreted as evidence for potentially interacting behavioral pathways rather than as proof of a single causal sequence. The domains reviewed here are not merely parallel predictors: instructors and peers may shape social opportunity and relatedness. These contextual conditions may influence perceived capability, group identity, motivation, and affective valuation, and these more proximal processes may influence whether participants continue attending (Michie et al., 2011; Rhodes & Kates, 2015; Teixeira et al., 2012).

However, existing syntheses have mainly focused on older adults in community exercise programs, program features that facilitate attendance, or the role of instructors in older-adult fitness, rather than providing a focused synthesis of psychosocial predictors across adult group fitness classes as a distinct literature (de Lacy-Vawdon et al., 2018; Farrance et al., 2016; Harvey & Griffin, 2020). Broader reviews of exercise adherence and physical-activity correlates have also aggregated heterogeneous clinical, community, and general physical-activity contexts, which limits their specificity for instructor-led group fitness classes delivered in commercial, university, and community contexts (Collado-Mateo et al., 2021; Schutzer, 2004; Trost et al., 2002). The resulting gap is therefore both empirical and conceptual: it remains unclear which theoretically linked psychosocial mechanisms have been examined as predictors of observed behavioral adherence in adult group fitness classes and whether the available evidence supports an integrated behavioral-science account rather than isolated construct-specific associations. Accordingly, the objective of this systematic review was to identify, critically appraise, and synthesize quantitative studies examining whether selected psychosocial constructs, organized as perceived-capability, social-relational/instructor, and affective-motivational mechanisms, are associated with behavioral adherence in adult instructor-led group exercise classes and programs.

## 2. Materials and Methods

This review was designed as a systematic review of quantitative evidence on psychosocial predictors of adherence to adult group fitness classes. The reporting framework follows the Preferred Reporting Items for Systematic Reviews and Meta-Analyses (PRISMA) 2020 statement, and the search methods are described in line with PRISMA-S (Rethlefsen et al., 2021). The systematic review protocol was registered in the Open Science Framework on 13 April 2026 with the ID number osf.io/5j73u.

### 2.1. Eligibility Criteria

Studies were eligible if they included adults aged 18 years or older, or populations explicitly described as adult, who participated in instructor-led group fitness or group exercise classes delivered in community, university, workplace, healthcare, or commercial fitness settings. Eligible class formats included aerobics and step aerobics, dance-fitness, indoor cycling, circuit or functional group training, yoga, Pilates, multicomponent community exercise classes, and analogous instructor-led group exercise modalities, provided that participation occurred in a shared class setting rather than exclusively through one-to-one training or home-based exercise.

The exposures of interest were psychosocial constructs hypothesized to influence adherence, specifically groupness or group cohesion, social support or relatedness, self-efficacy (including barrier self-efficacy), enjoyment or affective response, and instructor-related factors such as leadership, supervision, interpersonal style, autonomy-supportive or controlling behaviors, and coaching-related processes. Conceptually adjacent variables were eligible when they could be mapped onto one of these predefined domains. These domains were prespecified because they represent theoretically proximal determinants of repeated attendance within capability–opportunity–motivation and exercise-motivation frameworks: self-efficacy as perceived capability, groupness/cohesion and social support/relatedness as social opportunity and belonging, instructor behavior as a social-contextual determinant, and enjoyment/affective response as affective motivation (Rhodes & Kates, 2015; Teixeira et al., 2012).

The primary outcome was behavioral adherence to the class or program, defined as class attendance rate, number of sessions attended, retention or completion, persistence or maintenance, continued participation at follow-up, or dropout/attrition. Studies reporting only intention to continue exercising, without an accompanying behavioral adherence outcome, were excluded from the primary synthesis, although such variables could be extracted as contextual information when reported alongside adherence outcomes.

Quantitative observational studies, including analytical cross-sectional, cohort, prospective longitudinal, and other non-randomized association studies, were eligible. Randomized or non-randomized intervention studies were also eligible when they explicitly targeted one of the psychosocial domains of interest or reported an empirical association between a psychosocial construct and an adherence outcome. Mixed-methods studies were eligible only if extractable quantitative data relevant to the review question were reported separately. Qualitative studies, protocols, conference abstracts without full data, editorials, commentaries, narrative reviews, systematic reviews, dissertations not indexed as full reports in the searched databases, school physical education studies, competitive team-sport training studies, and purely individual personal-training or home-exercise studies were excluded.

### 2.2. Information Sources and Search Strategy

Electronic searches were conducted on 16 April 2026, in PubMed, Scopus, and Web of Science. No date limits were applied. No language filter was applied at the database-search stage. In addition to database searching, the reference lists of all included studies and of directly relevant review articles were searched to identify further eligible records. These three databases were selected to combine biomedical coverage, broad multidisciplinary citation coverage, and indexed literature from sport, health, and behavioral-science journals. We acknowledge that psychology-, sport-, and allied-health-specific databases such as PsycINFO, SPORTDiscus, and CINAHL were not searched. To mitigate this limitation, we supplemented database searching with manual backward reference checking of all included studies and directly relevant reviews, with particular attention to studies on group exercise adherence, group cohesion, self-efficacy, social support, instructor behavior, and enjoyment in exercise contexts. However, no additional eligible studies were identified through this manual reference-list process.

The search strategy was developed iteratively from the review question and from preliminary scoping of related reviews and primary studies on group exercise adherence, perceptions of groupness in fitness classes, social support in group exercise, self-regulation or self-efficacy in fitness clubs, and instructor behavior in exercise contexts. That scoping step was used to identify recurring terminology, spelling variants, and controlled vocabulary for three concept blocks: group fitness or class-based exercise, adherence or attendance, and psychosocial predictors of interest. The final strategies combined controlled vocabulary were available with free-text terms in titles, abstracts, and keywords and were adapted to the syntax of each database. The strategy intentionally prioritized sensitivity over specificity because group fitness studies are indexed inconsistently and may be described using broad setting terms, modality terms, or behavioral terms rather than a standardized group fitness label. Broad terms such as “gym,” “dance,” “participation,” and “satisfaction” were therefore retained to reduce the risk of missing eligible records, while specificity was imposed through the requirement that records satisfy all three concept blocks and subsequently meet strict eligibility criteria for adult instructor-led group exercise, eligible psychosocial predictors, and behavioral adherence outcomes.

[Title/abstract] “group exercise” OR “group fitness” OR “group-based exercise” OR “group-based physical activit*” OR “fitness class” OR “exercise class” OR “group class*” OR “exercise group*” OR “group training” OR “group session*” OR “community exercise class*” OR “health club*” OR “fitness club*” OR gym* OR aerobics OR yoga OR pilates OR dance OR “indoor cycling” OR spinning

AND

[Title/abstract] adher* OR attend* OR retention OR completion OR dropout OR attrition OR persist* OR continu* OR maintenance OR participation OR uptake OR “exercise behavior”

AND

[Title/abstract] “social support” OR “self efficacy” OR “self-efficacy” OR “interpersonal relations” OR groupness OR cohesion OR “group environment” OR “group climate” OR “peer support” OR companionship OR belonging OR relatedness OR “barrier self-efficacy” OR confidence OR enjoyment OR please OR pleasurable OR “affective response” OR “affective valence” OR fun OR satisfaction OR instructor* OR trainer* OR coach* OR leader* OR leadership OR supervision OR “autonomy support” OR “need support” OR “interpersonal behavior*” OR “motivational climate”

### 2.3. Selection Process

All records retrieved from the database searches were exported to Zotero (9.0.4, Corporation for Digital Scholarship, Vienna, Virginia, USA), and duplicates were removed through a combination of automated matching and manual verification. Two authors screened titles and abstracts against the predefined eligibility criteria. Full texts of potentially relevant records were then obtained and assessed independently by the same authors. Disagreements were resolved through discussion, with the support of a third author when consensus could not be reached. Reasons for exclusion at the full-text stage were documented.

### 2.4. Data Collection Process

A standardized data-extraction form was developed and piloted on a small sample of eligible studies before full extraction. Data were extracted independently by two authors, and discrepancies were resolved by discussion and, when required, by consultation with a third author.

### 2.5. Data Items and Outcomes

For each included study, the extracted data included authors, year of publication, country, study design, setting, recruitment approach, sample size, participant characteristics, class modality, class frequency and duration, intervention or follow-up length, psychosocial predictor domain, operational definition and measurement instrument for the predictor, timing of predictor assessment, operational definition of adherence, statistical methods, covariates included in adjusted models, and all effect estimates relevant to the review question.

The primary outcome set comprised behavioral adherence indicators, namely attendance proportion, number of sessions attended, retention or completion, persistence or maintenance, continued participation at follow-up, and dropout or attrition. When multiple adherence measures were reported within the same study, all were extracted. However, attendance-based measures were prioritized for cross-study comparison because they were expected to be the most frequently reported and the most directly comparable. Secondary contextual variables such as intention to continue exercising, satisfaction, or exercise identity were extracted only when they were reported alongside at least one behavioral adherence outcome.

### 2.6. Risk of Bias Assessment

Methodological quality and risk of bias were appraised with design-specific instruments. Comparative non-randomized intervention studies with ROBINS-I, and cohort studies with the revised JBI cohort tool. Risk-of-bias assessments were performed independently by two authors, with disagreements resolved by consensus or third-author support.

### 2.7. Effect Measures and Data Handling

For each study, associations between psychosocial predictors and adherence were extracted in the form most directly reported by the investigators, including odds ratios, risk ratios, hazard ratios, regression coefficients, correlation coefficients, mean differences in adherence across exposure groups, or intervention effect estimates. Before synthesis, we assessed whether domain-specific quantitative pooling was feasible, particularly for self-efficacy and groupness/cohesion, which were the most frequently represented predictor domains. A pooled estimate was considered appropriate only when studies provided a sufficiently comparable construct, behavioral adherence endpoint, follow-up window, and effect metric, or when conversion to a common metric could be performed without requiring strong model-dependent assumptions. This assessment was guided by the principle that meta-analysis should estimate a meaningful common parameter rather than merely produce a numerical average across statistically convertible but conceptually non-equivalent effects (Campbell et al., 2020).

Meta-analysis was not undertaken because the eligible studies used non-equivalent effect measures, different adherence endpoints, different follow-up windows, heterogeneous predictor instruments, and models with different covariate adjustment strategies. This limitation remained present within the self-efficacy domain. Self-efficacy was operationalized as barrier self-efficacy, scheduling self-efficacy, exercise-specific self-efficacy, or general exercise self-efficacy; adherence was operationalized as attendance proportion, number of sessions attended, later attendance, long-term attendance, or post-program continuation; and reported estimates included correlations, adjusted beta coefficients, hierarchical regression increments, structural-equation path coefficients, dichotomized mean differences, and negatively scored regression coefficients. In several cases, studies also reported domain-specific subscales or indirect pathway coefficients rather than a single comparable predictor–outcome effect. Pooling these estimates would therefore have required combining unadjusted and adjusted associations, direct and model-mediated effects, continuous and dichotomized predictors, and short- and longer-term adherence outcomes. Quantitative pooling under these conditions was judged likely to produce a statistical summary estimate with limited conceptual interpretability.

### 2.8. Synthesis Methods

A structured narrative synthesis was prespecified as the primary analytic approach because heterogeneity was verified in class modality, participant population, psychosocial construct operationalization, and adherence measurement. The synthesis approach followed principles consistent with Synthesis Without Meta-analysis guidance, in which transparent grouping, tabulation, comparison of effect direction and magnitude, and explicit explanation of non-pooling decisions are used when quantitative data are not sufficiently amenable to meta-analysis (Campbell et al., 2020). Included studies were grouped by predictor domain (groupness or cohesion, social support or relatedness, self-efficacy, enjoyment or affective response, and instructor-related factors) and by design, context, and population characteristics. Within each domain, evidence tables were used to summarize study characteristics, adherence definitions, effect direction, magnitude, statistical adjustment, and risk-of-bias judgments. To strengthen non-pooled quantitative interpretation, we additionally compared the reported magnitude and consistency of effects within each pathway using the original effect metrics reported by each study. Because effect metrics were not commensurable, this comparison was not used to generate a pooled effect estimate. Instead, effects were interpreted comparatively according to whether estimates were near-null, small, moderate, large, inconsistent, or model-dependent within their original analytic context. We also considered whether the association was direct or indirect, whether it remained evident in adjusted models, whether the predictor was temporally aligned with the adherence window, and whether the estimate came from a study with higher risk-of-bias concerns. We avoided simple vote counting based only on statistical significance and instead considered construct specificity, temporal alignment between predictor and adherence window, adjustment for confounding, risk of bias, and consistency of direction across studies. Where possible, we compared the direction, consistency, and strength of associations within each predictor domain while avoiding pooled estimates when the underlying constructs or adherence metrics were not sufficiently commensurable.

## 3. Results

### 3.1. Study Selection

**Figure 1:**
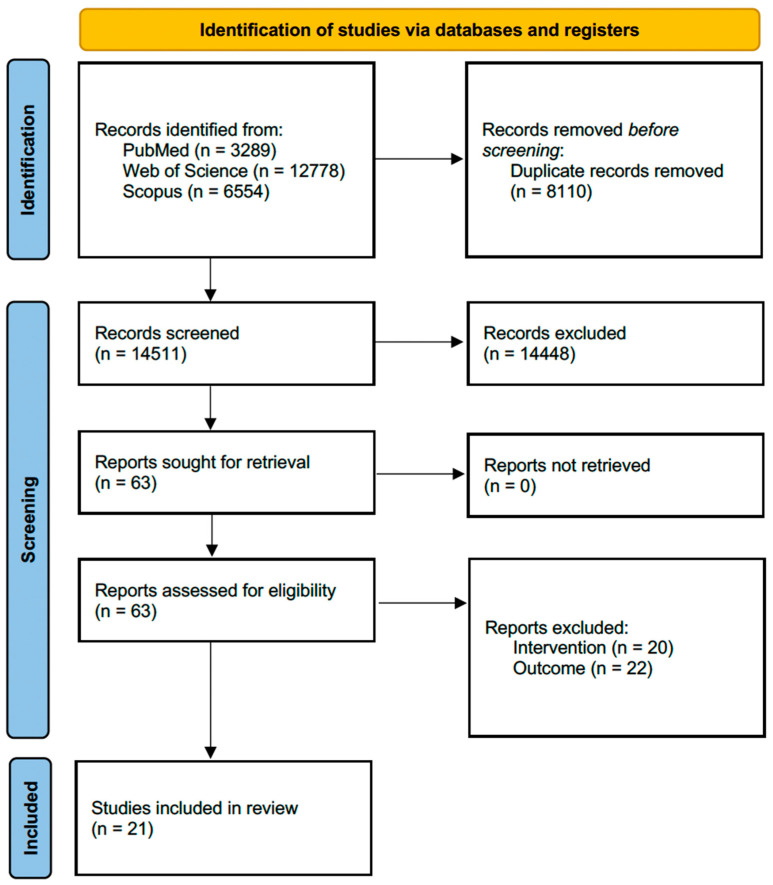
PRISMA flow diagram.

A total of 22,621 records were identified from PubMed (n = 3289), Web of Science (n = 12,778), and Scopus (n = 6554). Deduplication removed 8110 duplicates, leaving 14,511 unique records for title and abstract screening. Among these records, 63 reports were considered potentially eligible, excluding 14,448. Of those 63 reports, 42 were excluded and 21 were included. Figure 1 summarizes the process. The most frequent reasons for exclusion at full-text were absence of an eligible behavioral adherence outcome (n = 22) and lack of specificity to instructor-led group fitness or exercise classes (n = 20). Thus, the small final number of included studies primarily reflected the deliberately sensitive search strategy combined with narrow eligibility criteria, rather than an absence of broad search coverage. Many retrieved records addressed physical activity, exercise participation, satisfaction, or psychosocial constructs in general, but did not examine a behavioral adherence outcome within adult instructor-led group fitness or group exercise classes.

### 3.2. Characteristics of Included Studies

Characteristics of the included studies and evidence units are summarized in Table 1. Most studies were prospective cohort or longitudinal observational analyses, alongside two secondary adherence analyses nested within randomized trials, one analytical cross-sectional mediation study, and two non-randomized intervention studies. Most evidence units enrolled generally healthy adults, whereas four focused on clinical or rehabilitation populations. Table 1 presents the study characteristics, psychosocial exposure domains, and adherence outcomes extracted from each included study.

Methodological and fitness class characteristics showed substantial heterogeneity. Technique categories were most often mixed, focusing on instructor-led group fitness classes (n = 11), followed by multicomponent community exercise classes (n = 4), yoga (n = 3), aerobics or step aerobics (n = 3), and outpatient cardiac rehabilitation exercise groups (n = 1). Most evidence involved repeated or multi-session exposure, while five reflected ongoing membership or maintenance-phase participation in continuing classes.

When program length was reported, fixed-duration interventions typically lasted from 6 to 52 weeks, with a median reported duration of 12 weeks. Session duration, when stated, was usually 60 to 90 min. Predictor assessment was usually performed at baseline or early in programme participation and linked to subsequent attendance over short- or medium-term follow-up windows. Across studies, adherence was used mainly as attendance proportion or number of sessions attended. Much less often, studies examined continued participation at follow-up or dropout.

### 3.3. Risk of Bias Assessment and Critical Appraisal of Included Studies

Risk of bias assessment and critical appraisal results are presented in Table 2 and Table 3. Both non-randomized intervention studies were judged at serious risk of bias under ROBINS-I (Table 2). The main concerns were confounding, small selected samples, limited or unclear allocation procedures, and residual concerns about selective reporting. All JBI-appraised observational studies were judged to be of moderate quality, indicating some risk of bias rather than pervasive critical flaws. Common strengths were temporal ordering of psychosocial predictors before later adherence, objective or instructor-recorded adherence outcomes, and generally appropriate statistical analyses. The most recurrent limitations were failure to identify confounders or apply strategies to manage confounding, unclear or incomplete reporting of follow-up completeness, limited handling of incomplete follow-up, and occasional uncertainty about the validity of exposure measurement.

### 3.4. Synthesis of Findings by Higher-Order Behavioral Pathway

The findings were synthesized according to three higher-order behavioral pathways that reflect the conceptual structure of the review (Table 4): a cognitive/action-control pathway, a social-relational and instructor-shaped pathway, and an affective-motivational pathway. This synthesis is descriptive rather than causal because the included studies were heterogeneous in design, population, construct measurement, adherence definition, follow-up duration, and statistical adjustment. However, to reduce reliance on study-by-study reporting, we compared each pathway according to evidence volume, direction of association, reported effect magnitude, temporal alignment between predictor and adherence window, and whether the association was direct, indirect, adjusted, or model-dependent.

The cognitive/action-control pathway was represented primarily by self-efficacy, including barrier self-efficacy, scheduling self-efficacy, and exercise-specific self-efficacy. Across the included evidence, this was the most frequently examined individual-level pathway and was more often positively associated with attendance-related adherence than the other psychosocial domains. However, the pattern should be interpreted cautiously because not all studies reported significant associations, and positive findings were strongest when efficacy measures were behavior-specific and temporally aligned with the attendance window.

The social-relational and instructor-shaped pathway included groupness or cohesion, social support or relatedness, group identification, and instructor-related factors. Collectively, this pathway suggested that adherence may be more favorable when participants experience social attraction to the class, group identification, or supportive instructor behavior. However, broad cohesion and social support measures were not consistently associated with adherence, indicating that generic social climate may be less informative than specific indicators of social attachment, group identity, reliable alliance, or instructor-shaped motivational climate.

The affective-motivational pathway was represented by enjoyment and affective response. This was the least developed pathway in the eligible quantitative literature. The available studies suggest that affective constructs may be most informative when measured close to a specific attendance decision, such as anticipated enjoyment before the next class, whereas broader retrospective enjoyment measures were less consistently associated with adherence. Because few studies examined this pathway, the current evidence is insufficient to determine whether enjoyment is a weak predictor or an under-measured mechanism.

**Figure 2:**
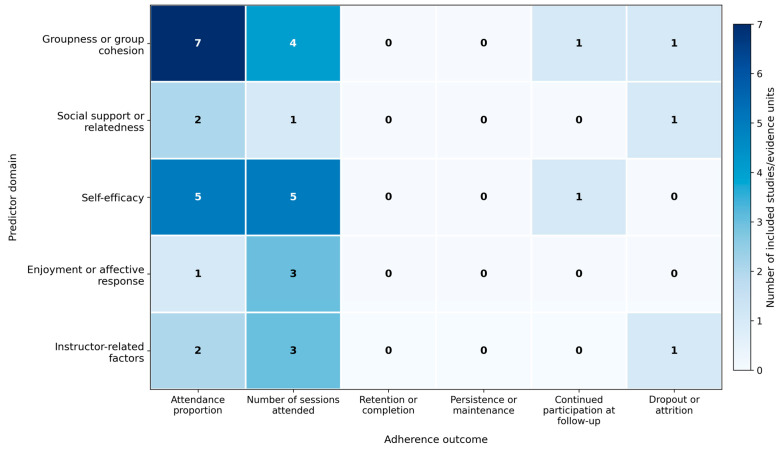
Evidence gap map.

The results indicate that the eligible literature is not evenly distributed across behavioral pathways. The strongest descriptive pattern concerns behavior-specific perceived capability, whereas social-relational and instructor-related evidence is suggestive but context-dependent, and affective-motivational evidence remains sparse. Table 5 provides the evidence underlying this pathway-level synthesis, and Figure 2 shows the distribution of evidence units across predictor domains and adherence outcomes.

Across pathways, the clearest quantitative signal was observed for self-efficacy and related perceived-capability constructs. Positive estimates in this domain were often in the small-to-moderate range when reported as correlations, standardized coefficients, or path coefficients, including class-attendance associations such as rs = 0.34, β = 0.34–0.40, and β = 0.383 in several studies. Larger effects were reported in selected subgroup or contrast-based analyses, such as a 28-percentage-point attendance difference between higher and lower self-efficacy groups, but these estimates were less directly comparable because of dichotomization, clinical context, or small samples. Null or weak findings were also present, particularly when exercise self-efficacy was not closely aligned with supervised class attendance, when participants were experienced exercisers, or when the outcome shifted from supervised attendance to post-program continuation. Thus, self-efficacy appears to be the most robust pathway, not because all studies were positive but because positive associations recurred across several independent settings and were strongest when the efficacy measure was behavior-specific and temporally aligned with the adherence outcome.

The social-relational and instructor-shaped pathway showed a more differentiated pattern. Broad cohesion and generic social support measures frequently produced near-null or small direct associations with attendance, whereas more specific constructs, including social attraction to the group, social cohesion, reliable alliance, group identification, and leader behavior, showed more favorable and sometimes moderate associations. For example, social attraction and group-integration indicators were generally more informative than undifferentiated cohesion, and group identification or leader-behavior measures showed favorable associations in studies that modeled instructor-shaped class climate. However, these effects were more context-dependent than self-efficacy effects, with some associations varying by membership duration, follow-up interval, class context, or statistical model.

The affective-motivational pathway had the weakest quantitative support. Anticipated enjoyment showed a small positive association with next-class attendance and short-term maintenance in one study, but this association attenuated when intention was included in the model. Retrospective enjoyment or broader enjoyment measures were not independent predictors in the other available studies. Therefore, the current evidence does not indicate that enjoyment is unimportant; rather, it suggests that affective predictors may require event-proximal measurement and repeated sampling designs to estimate their contribution to adherence reliably. Table 6 shows the comparative non-pooled synthesis of direction, magnitude, and robustness across behavioral pathways.

Although self-efficacy was the most frequently represented and most coherent psychosocial domain, formal pooling was not conducted after domain-specific feasibility assessment. Only a minority of self-efficacy studies reported direct r-type associations between self-efficacy and supervised class attendance. Other studies reported adjusted regression coefficients, hierarchical regression increments, structural-equation path coefficients, dichotomized self-efficacy contrasts, negatively scored regression coefficients, or associations with distinct adherence windows. Moreover, the self-efficacy construct differed across studies, including barrier self-efficacy, scheduling self-efficacy, exercise-specific self-efficacy, and general exercise self-efficacy. Adherence outcomes also differed, including attendance proportion, number of sessions attended, later attendance, long-term attendance, and post-program continuation. Because these estimates did not represent the same underlying predictor–outcome parameter, statistical conversion to a common metric would have produced a hybrid estimate with limited interpretability. The self-efficacy evidence was therefore synthesized narratively using direction, magnitude, construct specificity, adherence definition, temporal alignment, statistical adjustment, and risk of bias.

## 4. Discussion

This systematic review was undertaken to identify, critically appraise, and synthesize quantitative evidence on whether groupness or cohesion, social support or relatedness, self-efficacy, enjoyment or affective response, and instructor-related factors are associated with adherence to adult group fitness classes. Rather than interpreting these domains as isolated correlates, the findings are better understood as a theoretically patterned but methodologically heterogeneous evidence base. In behavioral terms, repeated attendance appears to depend on the interaction between perceived capability, social opportunity, motivational regulation, affective valuation, and the social structure of the class environment. This interpretation is consistent with the COM-B model, in which behavior is enabled by capability, opportunity, and motivation (Michie et al., 2011).

Within this integrated account, self-efficacy corresponds most closely to the perceived-capability and action-control components of adherence. Social Cognitive Theory positions self-efficacy as a central determinant of motivation and behavior because efficacy beliefs shape goal pursuit, persistence, and perceived ability to overcome environmental impediments (Bandura, 2004). The Theory of Planned Behavior provides a complementary interpretation because perceived behavioral control and intention may help explain how confidence-related beliefs are translated into attendance behavior (Armitage & Conner, 2001; Courneya & McAuley, 1995). Groupness, cohesion, social support, relatedness, and instructor behavior correspond more directly to social-contextual mechanisms. From a Self-Determination Theory perspective, instructors and peers may support or frustrate autonomy, competence, and relatedness, thereby influencing the quality of motivation for continued participation (Teixeira et al., 2012). From a group-dynamics perspective, social attraction, social integration, and group identification may make the class more than a repeated exercise exposure; they may transform attendance into participation in a meaningful social unit (P. A. Estabrooks & Carron, 1999; Fraser & Spink, 2002).

The main contribution of this review to behavioral science is therefore not simply that some psychosocial predictors are more consistently associated with attendance than others. Rather, the review suggests that adherence to instructor-led group fitness classes should be conceptualized as a multilevel behavioral process in which individual action-control beliefs operate within instructor-shaped and peer-shaped social environments. The theoretical contribution is best understood as a refinement of existing behavioral-maintenance frameworks for the specific ecology of instructor-led group exercise, not as a claim that a fully new adherence theory has been empirically established. In this refined account, repeated attendance is proposed to depend on a dynamic alignment between perceived capability, socially structured opportunity, affective valuation of the class experience, and the stabilizing effects of repeated participation (Michie et al., 2011; Rhodes & Sui, 2021). The strengthened non-pooled comparison of reported effect magnitudes supports this interpretation since self-efficacy showed the most recurrent small-to-moderate positive signal across studies, whereas social-relational and instructor-related factors showed more context-dependent effects and affective-motivational evidence remained sparse. This comparison does not imply that self-efficacy is the only important adherence mechanism. Rather, it indicates that the current quantitative literature measures perceived capability more frequently and more consistently than social, instructor-shaped, or affective mechanisms.

### 4.1. Self-Efficacy and Perceived Capability as the Clearest Evidence Cluster

Among the psychosocial domains, self-efficacy showed the most recurrent pattern of positive associations with attendance-related adherence, although the evidence should be interpreted cautiously because studies were heterogeneous, mostly observational, and varied in adjustment for confounding. Positive prospective findings were reported in university, community, and yoga-based programmes, with barrier self-efficacy, scheduling self-efficacy, and exercise-specific self-efficacy associated with better subsequent attendance in at least some settings (Bray et al., 2001; P. Estabrooks & Carron, 1998; McAuley et al., 2011; Shields et al., 2006; Speed-Andrews et al., 2012; Wininger, 2004; Yeung & Hemsley, 1997). Across these studies, the most informative measures were generally behaviour-specific and prospectively linked to an upcoming attendance window, suggesting that measurement specificity may partly explain why self-efficacy appeared more consistently related to adherence than broader psychosocial constructs. This suggests that perceived capability may function as a relatively proximal determinant of whether participants can translate class intentions into repeated attendance, particularly when the main barriers are logistical, self-regulatory, or confidence-related.

Theoretically, this pattern is most consistent with Social Cognitive Theory and the perceived-control component of the Theory of Planned Behavior. Self-efficacy should not be interpreted merely as a positive psychological trait; rather, it is a task-specific judgment about whether the participant can perform the target behavior under anticipated barriers (Bandura, 2004). In the context of group fitness classes, this means confidence in scheduling attendance, tolerating discomfort or fatigue, returning after missed sessions, and managing competing obligations. The comparatively more consistent pattern observed for behavior-specific and temporally proximal efficacy measures is compatible with a key theoretical point: confidence beliefs may be most informative when they are matched to the exact behavioral demand and decision window (Terry & O’Leary, 1995).

The self-efficacy findings were not uniformly positive, however, and the exceptions are informative. In a study (Bray et al., 2001), barrier self-efficacy predicted attendance among exercise initiates, whereas no self-efficacy subtype predicted attendance among experienced exercisers, suggesting that stage of behavioural adoption may modify the role of confidence beliefs. In another study (Dyrlund & Wininger, 2006), the adjusted coefficient for self-efficacy was positive but not statistically significant once other motivational constructs were considered. In a study (Hewett et al., 2019), exercise self-efficacy was not associated with attendance in a Bikram yoga sample, and in other research (Cheung et al., 2016), self-efficacy predicted intervention-period class attendance but not continued yoga practice after the structured classes had ended. These divergences suggest that the predictive value of self-efficacy depends somewhat on how closely the measure matches the behaviour of interest, whether the outcome concerns in-program attendance or post-program continuation, and whether participants are still in the early adoption phase. Even so, the overall direction of the literature was more coherent for self-efficacy than for any other psychosocial domain considered in the review. Therefore, the review should not be interpreted as establishing self-efficacy as a causal determinant of adherence. Rather, it indicates that self-efficacy is the domain for which the available association evidence is comparatively most recurrent, while the magnitude, independence, and causal direction of this relationship remain uncertain.

These inconsistencies should not be interpreted simply as contradictory findings. They may indicate boundary conditions of the self-efficacy theory. For exercise initiates, attendance may depend strongly on conscious self-regulation and confidence in overcoming barriers; for experienced exercisers, attendance may be more strongly supported by habit, identity, scheduling routines, or stable preferences. This distinction is important because physical-activity maintenance may involve a shift in the mechanisms that support behavior over time: early participation may rely more heavily on reflective control, barrier management, and perceived capability, whereas sustained participation may increasingly depend on routinization, contextual stability, identity, and reinforcement from prior successful participation (Rhodes & Sui, 2021). Similarly, confidence to attend a supervised class may not generalize to home practice after the class context is removed, because the target behavior changes from socially structured attendance to self-managed continuation. This distinction is important for behavioral theory because it suggests that self-efficacy effects are likely to be strongest when the efficacy measure, behavioral outcome, and environmental context are tightly aligned. The self-efficacy findings therefore refine, rather than merely confirm, self-efficacy theory since efficacy appears most predictive when it is behavior-specific, stage-specific, and matched to the attendance context being assessed.

### 4.2. Group Processes and Instructor-Related Factors as Relational Influences on Adherence

The evidence for groupness or cohesion was more heterogeneous, but it did not suggest that all dimensions of the group experience are equally relevant to adherence. Some studies reported null or weak direct associations between broad cohesion measures and attendance (Chermette et al., 2020; Courneya & McAuley, 1995; P. Estabrooks & Carron, 1999). However, more specific relational indicators, especially social attraction to the group, social integration, task integration over longer follow-up, and group identification, were more often associated with better adherence (Chermette et al., 2020; P. A. Estabrooks & Carron, 1999; Izumi et al., 2015; Loughead et al., 2001; Steffens et al., 2019). This tendency suggests that what matters may be less the presence of a generic cohesive atmosphere than the extent to which participants feel psychologically attached, socially embedded, or meaningfully connected to the class group.

This interpretation is consistent with Group Dynamics Theory, which treats cohesion as multidimensional rather than as a single global property of a group (P. A. Estabrooks & Carron, 1999). In group fitness contexts, social attraction may support attendance because participants want to return to people with whom they feel comfortable; social integration may support attendance because the class becomes a recognizable social unit; and task integration may matter when participants perceive shared goals, coordinated effort, or collective progress. The distinction between these dimensions helps explain why broad cohesion scores were less consistently predictive than more specific indicators of attachment, integration, or identification.

The instructor’s findings can also be interpreted more theoretically than as simple evidence that “good instructors” improve adherence. Instructors are social-contextual agents who may influence adherence through several pathways: they may strengthen competence by structuring achievable mastery experiences, support autonomy through non-controlling communication, foster relatedness through inclusion and recognition, and build group identity through shared language, norms, and routines. This interpretation links instructor behavior to Self-Determination Theory and group-dynamics mechanisms rather than treating instructor characteristics as isolated predictors (Christensen et al., 2006; Teixeira et al., 2012).

The variability across cohesion findings also points to contextual moderation. In a study (Chermette et al., 2020), social attraction to the group was positively associated with attendance only among participants with longer membership duration, indicating that the adherence relevance of group processes may depend on exposure time. In another study (P. A. Estabrooks & Carron, 1999), different cohesion dimensions appeared to matter at different follow-up intervals, with broader social and task integration associated with shorter-term adherence and task integration remaining relevant over longer follow-up. In two studies (Courneya & McAuley, 1995; P. Estabrooks & Carron, 1999), cohesion appeared more consistent with an indirect pathway through perceived control, attitudes, or intentions than with a strong direct effect on attendance. Thus, group processes may operate less as uniformly direct predictors and more as contextual influences that shape motivation and behavioural regulation over time.

These inconsistencies also suggest that group processes may have different functions across the adherence trajectory. Early in participation, social attraction and instructor warmth may reduce uncertainty and make return attendance less socially costly. As participation continues, group identification, task integration, and shared routines may become more important because the participant’s behavior is increasingly embedded in a recognizable social identity and class structure. This interpretation is consistent with social-identity approaches to physical activity, which propose that identification with an exercise group can make participation personally meaningful and behaviorally self-relevant (Stevens et al., 2018). Accordingly, null findings for broad cohesion or generic support measures may reflect poor theoretical targeting rather than the absence of a social mechanism: measures that do not distinguish attachment, identity, source of support, and timing may miss the specific relational process most relevant to attendance.

Instructor-related findings were similarly suggestive but conceptually broad. More favorable perceptions of instructor capability, identity leadership, leader enthusiasm, or health-promoter behavior were generally associated with better attendance or more consistent participation (Bray et al., 2001; Izumi et al., 2015; Loughead et al., 2001; Steffens et al., 2019). At the same time, a multilevel analysis (Hawley-Hague et al., 2014) showed that instructor-level influences are not uniformly positive and should not be reduced to a simple linear model. Some instructor characteristics were associated with better attendance, whereas others showed inverse associations, and the measured variables ranged from professional training to personality traits. Overall, the included studies support the view that instructor-related processes matter chiefly through the social climate they help to create, but the field still lacks a standardized framework for comparing these constructs across studies.

### 4.3. Social Support, Relatedness, and Enjoyment as Less Consistent Predictors

The evidence for social support or relatedness was weaker than might be expected from broader discussions of exercise participation. Direct associations with attendance were absent or very small in some studies (Courneya & McAuley, 1995), while one study found that reliable alliance was positively associated with better attendance and completion, but that guidance was inversely associated with adherence (Fraser & Spink, 2002). A study (Dyrlund & Wininger, 2006) reported an adjusted negative association between relatedness and attendance, but that finding should be interpreted cautiously because the relatedness subscale had weak psychometric properties and was modeled alongside other motivational constructs. A plausible interpretation is that generalized support measures may be too distal or too broad to predict class attendance consistently unless they capture specific, behaviourally relevant forms of support. The small number of eligible studies also means that the present review cannot determine whether the inconsistency reflects a weak relationship or a still-underdeveloped evidence base.

A theoretical interpretation is that social support and relatedness may often operate indirectly rather than as direct attendance predictors. In the Theory of Planned Behavior, social influences may affect adherence through attitudes, perceived behavioral control, subjective norms, and intention rather than through a simple direct pathway to behavior (Courneya & McAuley, 1995). In Self-Determination Theory, relatedness is also unlikely to be fully captured by generic support frequency; what matters is whether interpersonal interactions make participants feel respected, included, and volitionally engaged (Teixeira et al., 2012). Therefore, the weak direct evidence for broad support measures should not be interpreted as evidence that social context is unimportant. It more likely indicates that future studies need more theoretically precise measures of support quality, source, timing, and behavioral relevance.

The enjoyment findings can be interpreted similarly. Enjoyment may be most relevant when measured close to the attendance decision, because anticipated affect may influence whether participants intend to return to the next class (Feil et al., 2023). A study (Feil et al., 2025) reported that anticipated enjoyment predicted both next-class attendance and short-term maintenance, but these associations attenuated after intention was entered into the models, suggesting that intention may mediate part of the pathway from enjoyment to behavior. In contrast, enjoyment was not an independent predictor in two studies (Dyrlund & Wininger, 2006; Wininger, 2004). Retrospective global enjoyment, by contrast, may be too temporally diffuse to predict a specific attendance window. This helps explain why anticipated enjoyment appeared more predictive than broader retrospective enjoyment measures and suggests that affective constructs should be modeled as time-sensitive motivational processes rather than as stable background preferences.

### 4.4. Integrative Theoretical Contribution to Behavioral Science

The findings support a provisional integrative model of behavioral adherence to adult group fitness classes. The model extends the Introduction’s COM-B mapping by treating capability, opportunity, and motivation as interacting processes that may change across repeated attendance episodes rather than as fixed predictor categories (Michie et al., 2011). In this model, self-efficacy and perceived behavioral control represent an individual action-control pathway. Social attraction, cohesion, relatedness, and group identification represent a social-belonging pathway; instructor behavior represents an upstream social-contextual pathway; and enjoyment or affective response represents an affective-motivational pathway. These pathways should be viewed as complementary rather than competing explanations. For example, an instructor may strengthen perceived competence and self-efficacy, create a more need-supportive climate, and facilitate group identity; these social-contextual processes may then influence intention, perceived control, affective valuation, and repeated attendance. Conversely, successful attendance episodes may reinforce later self-efficacy, anticipated enjoyment, perceived belonging, and class identity, whereas missed sessions or negative class experiences may weaken these same processes and increase the likelihood of disengagement (McAuley & Blissmer, 2000; Rhodes & Kates, 2015; Rhodes & Sui, 2021).

**Figure 3:**
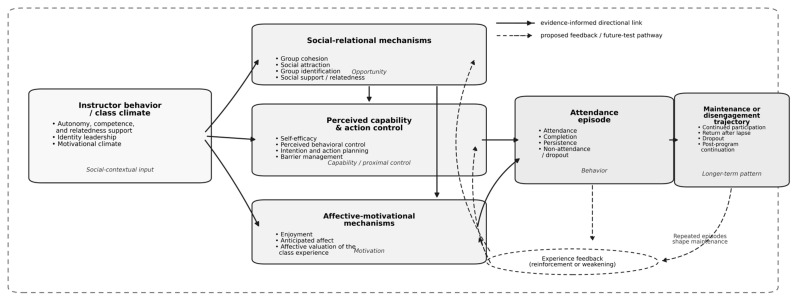
Proposed dynamic behavioral-process model of adherence to adult group fitness classes.

This synthesis contributes to behavioral science theory by showing that group fitness adherence is not adequately explained by individual motivation alone or by social atmosphere alone. The evidence is most compatible with a multilevel account in which behavior-specific confidence operates within a social environment that can either support or weaken motivation. This interpretation also helps reconcile apparently inconsistent findings. Self-efficacy may be the most consistent predictor because it is proximal to the attendance decision, whereas cohesion, social support, instructor behavior, and enjoyment may be more sensitive to timing, context, measurement specificity, and whether their effects are modeled directly or indirectly (Figure 3). This interpretation should be understood as theory-informed refinement rather than definitive theory testing. The included studies do not allow formal estimation of reciprocal pathways, longitudinal mediation, class-level clustering, or maintenance trajectories. Nevertheless, by specifying plausible feed-forward links among instructor-shaped opportunity, social belonging, perceived capability, affective valuation, intention, and repeated attendance, the review moves beyond a variable-summary model toward a provisional behavioral adherence mechanism that can be tested in future longitudinal and multilevel studies (Feil et al., 2023; Michie et al., 2011; Rhodes & Sui, 2021).

The model’s contribution is therefore not the replacement of COM-B, Social Cognitive Theory, Self-Determination Theory, or social-identity approaches, but their integration within the specific behavioral ecology of instructor-led group exercise. In this ecology, adherence is not only an individual decision to exercise; it is a repeated decision to return to a socially organized class at a particular time, with particular people, under the influence of instructor behavior, prior attendance experiences, anticipated affect, and perceived ability to manage barriers. This distinction expands the behavioral-maintenance interpretation by specifying why predictors may change in relevance across the trajectory from initial attendance to continued participation: perceived capability may be especially salient when participants are still managing barriers, social identity and class routines may become more salient as the class becomes part of the participant’s social world, and affective valuation may operate most strongly when measured close to a specific attendance opportunity (Michie et al., 2011; Rhodes & Sui, 2021; Teixeira et al., 2012).

Future research should therefore move from single-predictor models toward theory-based longitudinal designs that test mediation and moderation across levels. Studies should examine whether instructor behavior predicts attendance through perceived competence, autonomy support, relatedness, group identification, or enjoyment; whether self-efficacy mediates the relation between early mastery experiences and subsequent attendance; and whether group cohesion affects adherence directly or through attitudes, perceived behavioral control, and intention. Multilevel designs are particularly important because participants are nested within classes and instructors, and several relevant mechanisms operate at the class or group level rather than only at the individual level.

### 4.5. Why Were Findings Inconsistent? Methodological and Theoretical Sources of Heterogeneity

Several features of the evidence base help explain why the findings were more consistent in some domains than in others. The inconsistencies were not random. They appear to reflect at least five recurring sources of heterogeneity. Several studies used broad construct labels that may not have captured the theoretically active mechanism. For example, generic social support or global cohesion may be less predictive than social attraction, group identification, reliable alliance, or instructor-shaped motivational climate because these latter constructs are more directly linked to the decision to return to a specific class. Moreover, predictor timing differed across studies. Constructs measured close to an attendance decision, such as anticipated enjoyment or behavior-specific self-efficacy, may be more predictive than retrospective or global evaluations measured outside the relevant decision window. Additionally, adherence endpoints were not equivalent since attending supervised classes, returning after missed sessions, completing a program, and continuing after program withdrawal are related but theoretically distinct behaviors. Participant stage may have modified effects, because exercise initiates may depend more on self-regulatory confidence, whereas experienced participants may depend more on habit, identity, class routines, and stable preferences. Finally, several mechanisms may operate indirectly or at different levels of analysis, so direct individual-level associations may underestimate instructor-level, class-level, or mediated social effects.

Most studies used prospective observational designs, which are stronger than cross-sectional analyses for establishing temporal ordering but remain vulnerable to residual confounding. Most observational studies were judged to have some risk of bias, especially because confounders were often not clearly identified or adequately handled, and follow-up completeness or management of attrition was frequently imperfect. The recurrent associations observed in the review are therefore best interpreted as signals of likely relevance rather than as evidence of causal effect.

Substantial heterogeneity was also present in class modality, population, programme duration, predictor measurement, and outcome definition. Many studies examined mixed or incompletely specified instructor-led classes rather than clearly delimited modalities, which limits precision when interpreting psychosocial influences by exercise type. Likewise, the outcome domain was dominated by attendance proportion or number of sessions attended, whereas continued participation at follow-up and dropout were much less frequently studied, and no standardized body of evidence was available for retention or persistence as distinct behavioural endpoints. This concentration on attendance outcomes is understandable because attendance is observable and readily recorded, but it narrows the field’s ability to distinguish determinants of programme initiation, short-term compliance, and longer-term maintenance.

The evidence base was also concentrated in university and community contexts, often with female-predominant samples and, in several cases, older adults. Some of the most informative cohesion studies were conducted in older-adult community exercise programmes (P. A. Estabrooks & Carron, 1999; P. Estabrooks & Carron, 1998; Loughead et al., 2001), whereas several self-efficacy studies were based in university or structured programme settings (Bray et al., 2001; Dyrlund & Wininger, 2006; Shields et al., 2006). Apparent differences between psychosocial domains may therefore reflect setting and population as well as theory.

### 4.6. Limitations of the Evidence Base, Review Limitations, and Future Research

Several psychosocial domains were represented by only a small number of studies, and even within the better-represented domains, constructs with similar labels were not always operationalized in comparable ways. The literature also rarely examined multiple psychosocial domains within the same well-adjusted longitudinal framework, making it difficult to determine whether self-efficacy, group identification, cohesion, instructor behavior, and enjoyment contribute independently or through overlapping mechanisms. Consequently, the dynamic process model proposed in this review should be interpreted as a theoretically grounded synthesis of the available evidence rather than as an empirically confirmed causal pathway; the current literature does not yet provide sufficiently repeated, temporally aligned, or multilevel data to test reciprocal effects, mediation, or maintenance mechanisms directly. Future studies would be strengthened by clearer theoretical models, more explicit construct definitions, and validated measures that are tightly matched to the target behaviour and time window. Moreover, a limitation of this review is that the electronic search was restricted to PubMed, Scopus, and Web of Science. Although these databases provide broad biomedical and multidisciplinary coverage, the absence of PsycINFO, SPORTDiscus, and CINAHL may have reduced the retrieval of studies indexed primarily in psychology, sport science, exercise science, rehabilitation, nursing, or allied-health databases. We attempted to mitigate this limitation through a sensitive search strategy, no date or language restriction at the database-search stage, and manual reference-list checking of included studies and relevant reviews. Nevertheless, the possibility that some eligible studies were missed cannot be excluded.

A second major limitation is the narrow outcome architecture of the field. Most evidence concerns short-term attendance rather than retention, persistence, or maintenance after structured programming ends. This matters because the psychosocial processes that predict whether someone attends the next scheduled class may differ from those that support continued participation across months or after programme withdrawal. Future research should therefore distinguish more clearly between attendance, completion, dropout, and longer-term continuation, and should measure these endpoints consistently enough to permit more informative between-study comparisons and, where appropriate, future meta-analysis. Although exploratory meta-analysis was considered, including within the self-efficacy domain, the available studies did not provide sufficiently comparable effect estimates, predictor definitions, adherence outcomes, follow-up windows, or model specifications to support a meaningful pooled estimate. This was not only a statistical limitation but a conceptual one: the available self-efficacy estimates did not represent a single common estimand. Pooling them would have required combining barrier, scheduling, exercise-specific, and general self-efficacy measures; attendance proportion, session count, later attendance, and post-program continuation outcomes; and correlation, regression, path, R^2^ increment, and dichotomized mean-difference estimates. Therefore, the synthesis prioritized structured comparison of direction, magnitude, study context, temporal alignment, adjustment strategy, and risk of bias rather than statistical aggregation. Future meta-analysis may become appropriate if primary studies use more standardized self-efficacy measures, report directly comparable behavioral adherence outcomes, provide sufficient effect-size information, and distinguish short-term attendance from longer-term maintenance.

A third limitation is the weakness of the causal evidence. Most studies were prospective cohort or longitudinal observational analyses, many samples were modest, and adjustment for confounding was limited. Stronger evidence will require adequately powered longitudinal studies with prespecified confounder sets, repeated measurement of psychosocial constructs, and clearer attention to mediation and moderation. Future intervention studies should also move beyond small or weakly controlled team-building pilots and examine whether changes in self-efficacy, group identification, or instructor interpersonal style translate into measurable adherence gains under lower-risk designs.

### 4.7. Practical Implications

The practical implication of the theoretical synthesis is that adherence strategies should target mechanisms rather than only class exposure. A Social Cognitive Theory-informed strategy would emphasize mastery experiences, barrier planning, and confidence to attend under realistic constraints. A Self-Determination Theory-informed strategy would emphasize autonomy-supportive communication, competence support, and relatedness. A Theory of Planned Behavior-informed strategy would target attitudes, perceived behavioral control, and intention formation. A group-dynamics-informed strategy would develop social attraction, shared identity, and task integration within the class. These approaches are not mutually exclusive. In group fitness settings, they are likely to be most useful when integrated.

Although the evidence does not support definitive causal recommendations, it suggests several provisional directions for class design and participant support. Efforts to improve adherence may be more productive when they focus on strengthening participants’ confidence that they can attend consistently, overcome barriers, and manage the practical demands of participation, rather than when they rely on nonspecific encouragement alone. This emphasis is consistent with the comparatively recurrent evidence for behaviour-specific self-efficacy, but it should be regarded as a provisional practice implication rather than a definitive causal recommendation (Bray et al., 2001; P. Estabrooks & Carron, 1998; McAuley et al., 2011; Shields et al., 2006; Wininger, 2004). In practical terms, that may include barrier-planning support, scheduling routines, graduated mastery experiences, and early success experiences during the first weeks of enrolment.

The findings also suggest that the social environment of the class should not be treated as incidental. Social attraction to the group, social integration, group identification, and supportive leader behaviors were more consistently linked to adherence than were broad or undifferentiated cohesion scores. For practice, this implies that instructors and programme designers may wish to prioritize strategies that foster recognizable group identity, interpersonal familiarity, and meaningful participant-to-participant connection, while also maintaining competent, motivating, and responsive leadership (Izumi et al., 2015; Loughead et al., 2001; Steffens et al., 2019). At the same time, the mixed evidence for social support and enjoyment indicates that practitioners should be cautious about assuming that any positive class atmosphere will automatically translate into sustained attendance. Measurement and intervention development should instead focus on the specific psychosocial mechanisms most closely tied to class participation decisions.

## 5. Conclusions

Across a relatively small, heterogeneous, and largely observational evidence base, self-efficacy was the psychosocial domain most frequently reported as positively associated with attendance-related adherence in adult instructor-led group exercise settings, although this pattern remains vulnerable to residual confounding, measurement heterogeneity, and differences in adherence operationalization. Theoretical interpretation of the inconsistencies suggests that adherence may be best understood as a dynamic maintenance process in which the dominant mechanisms vary according to behavioral stage, measurement timing, social context, and whether the outcome concerns supervised attendance or longer-term continuation. Evidence for group-related and instructor-related factors was suggestive but context-dependent, whereas evidence for social support/relatedness and enjoyment remained limited and inconsistent.

The central behavioral-science message of this review is that adherence to group fitness classes should not be interpreted as a simple expression of individual willpower or motivation. Rather, repeated attendance appears to emerge through interacting cognitive, motivational, affective, and social mechanisms. Participants must believe that they can attend despite barriers, experience the class as socially meaningful, respond to an instructor-shaped motivational climate, and attach positive value to continued participation. This synthesis therefore positions exercise adherence as a multilevel behavioral process involving perceived capability, social belonging, instructor-shaped motivational climate, and affective valuation. At the field level, this shifts the interpretation of group fitness adherence from a question of whether adults “like” or “intend” to attend classes toward a more precise question of which mechanisms make repeated attendance easier, more meaningful, more rewarding, and more sustainable. For practice, the findings suggest that adherence strategies should not rely only on class availability, general encouragement, or positive atmosphere. Instead, programme designers and instructors should consider mechanism-targeted strategies that strengthen barrier-specific confidence, create early mastery experiences, foster recognizable group identity, support participant-to-participant connection, and maintain an autonomy-supportive and competence-supportive instructional climate.

By linking Social Cognitive Theory, the Theory of Planned Behavior, Self-Determination Theory, and Group Dynamics Theory within a single account of repeated class attendance, the review provides a conceptual framework for understanding why adults continue or discontinue participation in group fitness settings. However, stronger explanatory claims require more conceptually consistent and methodologically rigorous research. Future studies should move beyond single-level predictor models and use longitudinal, multilevel, and causally informative designs that account for participant nesting within classes and instructors, distinguish individual-level perceptions from class-level or instructor-level effects, and model whether instructor behavior influences adherence through perceived competence, autonomy support, relatedness, group identification, enjoyment, or self-efficacy. Research on affective mechanisms should use ecological momentary assessment or other intensive repeated-measurement approaches to capture anticipated enjoyment, affective valence, and post-class affect close to the attendance decision rather than relying only on retrospective global enjoyment ratings. The next stage of this literature should therefore test dynamic adherence pathways using standardized behavioral adherence outcomes, validated construct-specific measures, prespecified confounder sets, instructor- and class-level clustering, repeated affective assessment, and longitudinal mediation or causal pathway models capable of distinguishing short-term attendance from longer-term maintenance.

## Figures and Tables

**Table 1:** Characteristics of included studies examining psychosocial predictors of adherence to adult group fitness classes.

Study	Population (n Analyzed)	Design	Class Modality/Technique	Predictor Domain(s)	Adherence Outcome(s)
(Bray et al., 2001)	Healthy adults (n = 127)	Prospective cohort or longitudinal observational	Mixed or unclear instructor-led group fitness class: Several structured instructor-led group fitness classes; exact modalities not clearly reported	Self-efficacy; Instructor-related factors	Attendance proportion
(Chermette et al., 2020)	Adults with chronic heart diseases participating in phase III outpatient cardiac rehabilitation exercise groups (n = 474)	Prospective cohort or longitudinal observational	Outpatient cardiac rehabilitation exercise group: Outpatient cardiac rehabilitation exercise group with diverse physical activities, relaxation, and healthy lifestyle information	Groupness or group cohesion	Number of sessions attended
(Cheung et al., 2016)	Older women with knee osteoarthritis living in the community (n = 34)	Randomized controlled trial	Yoga: Hatha yoga program combining weekly group classes and prescribed home practice	Self-efficacy	Number of sessions attended; Continued participation at follow-up
(Courneya & McAuley, 1995)	Adult participants in structured university exercise classes (n = 62)	Prospective cohort or longitudinal observational	Aerobics and step aerobics: University aerobics program with multiple exercise classes	Social support or relatedness; Groupness or group cohesion	Attendance proportion
(Dyrlund & Wininger, 2006)	Undergraduate students in university group fitness classes (n = 189)	Prospective cohort or longitudinal observational	Mixed or unclear instructor-led group fitness class: Abdominal conditioning, kickboxing, step aerobics, and yoga classes	Self-efficacy; Enjoyment or affective response; Social support or relatedness	Number of sessions attended
(P. Estabrooks & Carron, 1998)	Older adult exercisers in university-based exercise classes (n = 157)	Prospective cohort or longitudinal observational	Mixed or unclear instructor-led group fitness class: Power walking, strength training, cardiovascular training, and Tai Chi classes	Self-efficacy	Attendance proportion
(P. A. Estabrooks & Carron, 1999)	Older adult exercisers in continuing group exercise classes (n = 69)	Prospective cohort or longitudinal observational	Mixed or unclear instructor-led group fitness class: Exercise classes ranging from aerobic fitness to strength training to improved flexibility	Groupness or group cohesion	Attendance proportion
(P. Estabrooks & Carron, 1999)	Older adult exercisers in university-based classes (n = 179)	Prospective cohort or longitudinal observational	Mixed or unclear instructor-led group fitness class: Power walking, strength training, cardiovascular training, and Tai Chi classes	Groupness or group cohesion	Attendance proportion
(Feil et al., 2025)	Adults in weekly exercise classes across university and sports-club settings (n = 363)	Prospective cohort or longitudinal observational	Mixed or unclear instructor-led group fitness class: Weekly fitness classes and non-competitive sports classes including yoga, Pilates, Zumba, full-body workouts, badminton, volleyball, and basketball	Enjoyment or affective response	Attendance proportion; Number of sessions attended
(Fraser & Spink, 2002)	Adult women instructed to exercise for prevention or rehabilitation of chronic disease risk or chronic conditions (n = 49)	Prospective cohort or longitudinal observational	Mixed or unclear instructor-led group fitness class: Light stretching warm-up, individualized light aerobic activity such as walking, stationary cycling, or stationary rowing, followed by light calisthenics and stretching	Social support or relatedness; Groupness or group cohesion	Attendance proportion; Dropout or attrition
(Hawley-Hague et al., 2014)	Older adults in community exercise classes, many with long-term conditions (n = 189)	Prospective cohort or longitudinal observational	Multicomponent community exercise class: Multicomponent community exercise classes for older adults including aerobic, strength, balance, and stretching components; classes could be mostly seated or mostly standing	Groupness or group cohesion; Instructor-related factors	Number of sessions attended; Dropout or attrition
(Hewett et al., 2019)	Stressed and sedentary adults without diagnosed chronic disease (n = 29)	Randomized controlled trial	Yoga: Standardized Bikram yoga classes in a heated room using a fixed sequence of postures and scripted instruction	Self-efficacy	Number of sessions attended
(Izumi et al., 2015)	Racially and ethnically diverse community adults from low-to-moderate income Detroit neighborhoods participating in a heart-health walking program (n = 603)	Prospective cohort or longitudinal observational	Multicomponent community exercise class: Structured community walking group sessions including warm-up, neighborhood walking, and cool-down	Instructor-related factors; Groupness or group cohesion	Number of sessions attended
(Loughead et al., 2001)	Older adult exercisers in recreational activity classes (n = 117)	Analytical cross-sectional	Mixed or unclear instructor-led group fitness class: Recreational group exercise classes including clogging, aerobic exercise, line dancing, tai chi, and power walking	Instructor-related factors; Groupness or group cohesion	Attendance proportion
(McAuley et al., 2011)	Inactive community-dwelling older adults without medical contraindications to exercise (n = 177)	Prospective cohort or longitudinal observational	Mixed or unclear instructor-led group fitness class: Walking group classes or flexibility, toning, and balance group classes	Self-efficacy	Attendance proportion
(Shields et al., 2006)	Adult exercisers in structured exercise classes, mostly already intermittently or regularly active in the previous year (n = 260)	Prospective cohort or longitudinal observational	Mixed or unclear instructor-led group fitness class: Structured exercise classes across community, university, and private fitness facilities; specific class modalities were not fully described.	Self-efficacy	Attendance proportion
(Speed-Andrews et al., 2012)	Post-adjuvant-therapy breast cancer survivors in the survivorship phase and disease-free at enrollment (n = 23)	Prospective cohort or longitudinal observational	Yoga: Iyengar yoga classes using props and pose modifications for breast cancer survivors	Self-efficacy	Attendance proportion
(Steffens et al., 2019)	Adults attending ongoing group exercise classes in commercial fitness settings (n = 249)	Prospective cohort or longitudinal observational	Mixed or unclear instructor-led group fitness class: Mixed group exercise modalities including cardiorespiratory, strength-conditioning, yoga, and Pilates classes	Groupness or group cohesion; Instructor-related factors	Number of sessions attended
(Watson et al., 2004)	Very old adults in a residential seniors’ facility exercise class who passed cognitive screening (n = 11)	Non-randomized intervention	Multicomponent community exercise class: Stretch and Strengthen class focused on strength, balance, coordination, and flexibility	Groupness or group cohesion	Attendance proportion
(Wininger, 2004)	Full-time female university students in not-for-credit step-aerobics classes (n = 71)	Prospective cohort or longitudinal observational	Aerobics and step aerobics: Step-aerobics classes with stepping activity, warm-up, prolonged aerobic workout, and cool-down	Self-efficacy; Enjoyment or affective response	Number of sessions attended
(Yeung & Hemsley, 1997)	Initially sedentary adult women without psychiatric disorder, obesity, or exercise-limiting medical conditions (n = 46)	Prospective cohort or longitudinal observational	Aerobics and step aerobics: Supervised aerobics program with warm-up, aerobic choreography, floor work, and cool-down	Self-efficacy	Number of sessions attended

**Table 2:** Risk of bias of non-randomized intervention studies using ROBINS-I.

Study	Bias Due to Confounding	Bias in Selection of Participants	Bias in Classification of Interventions	Bias Due to Deviations from Intended Interventions	Bias Due to Missing Data	Bias in Measurement of Outcomes	Bias in Selection of Reported Results	Overall Risk of Bias
(P. A. Estabrooks & Carron, 1999)	Serious	Moderate	Low	Moderate	Low	Low	Moderate	Serious risk of bias
(Watson et al., 2004)	Serious	Serious	Low	Moderate	Serious	Low	Moderate	Serious risk of bias

**Table 3:** Critical appraisal of observational studies using the JBI cohort tool.

Study	Groups Similar and Recruited from Same Population	Exposures Measured Similarly	Exposure Measured Validly	Confounders Identified	Strategies to Deal with Confounders	Participants Free of Outcome at Start	Outcomes Measured Validly	Follow-Up Time Reported and Sufficient	Follow-Up Complete or Explained	Strategies for Incomplete Follow-Up	Appropriate Statistical Analysis	Overall Appraisal
(Bray et al., 2001)	Yes	Yes	Unclear	No	No	Yes	Yes	Yes	Unclear	No	Yes	Moderate quality/some risk of bias
(Chermette et al., 2020)	Yes	Yes	Unclear	No	No	Yes	Yes	Yes	Unclear	No	Yes	Moderate quality/some risk of bias
(Cheung et al., 2016)	Yes	Yes	Yes	No	No	Yes	Yes	Yes	Yes	Yes	Yes	Moderate quality/some risk of bias
(Courneya & McAuley, 1995)	Yes	Yes	Yes	No	No	Yes	Yes	Yes	Yes	Yes	Yes	Moderate quality/some risk of bias
(Dyrlund & Wininger, 2006)	Yes	Yes	Yes	No	No	Yes	Yes	Yes	No	No	Yes	Moderate quality/some risk of bias
(P. Estabrooks & Carron, 1998)	Yes	Yes	Unclear	No	No	Yes	Yes	Yes	Yes	No	Yes	Moderate quality/some risk of bias
(Bray et al., 2001)	Yes	Yes	Unclear	No	No	Yes	Yes	Yes	Yes	No	Yes	Moderate quality/some risk of bias
(P. Estabrooks & Carron, 1998)	Yes	Yes	Yes	No	No	Yes	Yes	Yes	Yes	No	Yes	Moderate quality/some risk of bias
(P. Estabrooks & Carron, 1999)	Yes	Yes	Yes	No	No	Yes	Yes	Yes	Unclear	No	Yes	Moderate quality/some risk of bias
(Feil et al., 2025)	Yes	Yes	Yes	No	No	Yes	Yes	Yes	Unclear	No	Yes	Moderate quality/some risk of bias
(Fraser & Spink, 2002)	Yes	Yes	Unclear	No	No	Yes	Yes	Yes	Yes	Yes	Yes	Moderate quality/some risk of bias
(Hawley-Hague et al., 2014)	Yes	Yes	Yes	Yes	Yes	Yes	Yes	Yes	Unclear	No	Yes	Moderate quality/some risk of bias
(Hewett et al., 2019)	Yes	Yes	Yes	No	No	Yes	Yes	Yes	Yes	Yes	Yes	Moderate quality/some risk of bias
(Izumi et al., 2015)	Yes	Yes	Yes	Yes	Yes	Unclear	Yes	Yes	Unclear	No	Yes	Moderate quality/some risk of bias
(Loughead et al., 2001)	Yes	Yes	Yes	No	No	Unclear	Yes	Unclear	Unclear	No	Yes	Moderate quality/some risk of bias
(McAuley et al., 2011)	Yes	Yes	Yes	No	No	Unclear	Yes	Yes	Unclear	No	Yes	Moderate quality/some risk of bias
(Shields et al., 2006)	Yes	Yes	Yes	No	No	Unclear	Yes	Yes	Unclear	No	Yes	Moderate quality/some risk of bias
(Speed-Andrews et al., 2012)	Yes	Yes	Unclear	No	No	Yes	Yes	Yes	Yes	No	Yes	Moderate quality/some risk of bias
(Steffens et al., 2019)	Yes	Yes	Yes	No	No	Unclear	Unclear	Yes	Unclear	No	Yes	Moderate quality/some risk of bias
(Wininger, 2004)	Yes	Yes	Yes	No	No	Unclear	Yes	Yes	Unclear	No	Yes	Moderate quality/some risk of bias
(Yeung & Hemsley, 1997)	Yes	Yes	Unclear	No	No	Yes	Unclear	Yes	Unclear	No	Yes	Moderate quality/some risk of bias

**Table 4:** Higher-order synthesis of psychosocial pathways associated with adherence to adult group fitness classes.

Higher-Order Pathway	Constructs Mapped to Pathway	Evidence Coverage	Collective Pattern Across Studies	Main Adherence Outcomes Represented	Interpretation and Caution
Cognitive/action-control pathway	Self-efficacy, including barrier self-efficacy, scheduling self-efficacy, and exercise-specific self-efficacy; perceived behavioral control when reported in theory-based models.	Self-efficacy was represented in 10 studies across university, community, yoga, aerobics, and older-adult exercise contexts.	This pathway showed the most recurrent pattern of positive associations with attendance-related adherence. Positive findings were more apparent when the efficacy measure was behavior-specific and prospectively aligned with the attendance window. However, null or weaker findings were also reported, particularly when the outcome shifted from supervised class attendance to post-program continuation or when experienced exercisers were analyzed separately.	Attendance proportion; number of sessions attended; continued participation at follow-up.	Perceived capability may be a relatively proximal action-control mechanism for repeated attendance decisions. This should be interpreted as comparative consistency rather than causal evidence because studies were heterogeneous, mostly observational, and varied in adjustment for confounding.
Social-relational and instructor-shaped pathway	Groupness, cohesion, social attraction, social integration, group identification, social support, relatedness, instructor efficacy, leader behavior, motivational training, and identity leadership.	Groupness/cohesion was represented by 11 studies; social support/relatedness by 3 studies; and instructor-related factors by 5 studies, with overlap across domains.	The collective pattern was suggestive but mixed. More favorable associations were reported for social attraction to the group, social cohesion, reliable alliance, group identification, leader behavior, and some instructor-related constructs. Broad cohesion, generic social support, and some task-focused indicators were less consistently related to adherence, and some effects appeared conditional on membership duration, follow-up interval, or model specification.	Attendance proportion; number of sessions attended; dropout or attrition; continued participation after a program hiatus.	The evidence suggests that social belonging and instructor-shaped motivational climate may contribute to adherence, but these influences appear context-dependent and measurement-sensitive. Future studies should distinguish individual-level social perceptions from class-level or instructor-level effects and should account for clustering by class or instructor.
Affective-motivational pathway	Enjoyment, anticipated enjoyment, retrospective enjoyment, affective response, and affective valence.	Eligible quantitative evidence was limited to 3 studies.	Evidence was sparse and inconsistent. Anticipated enjoyment assessed close to the next attendance decision showed small positive associations with next-class attendance and short-term maintenance, but these associations attenuated when intention was modeled. Retrospective or broader enjoyment measures were not independent predictors in the available studies.	Attendance proportion; number of sessions attended; short-term maintenance over subsequent classes.	Affective experience may operate as a time-sensitive motivational or reinforcement process, but the current evidence is insufficient for firm conclusions. Future studies should use repeated or event-proximal affect measures linked to specific attendance windows.

**Table 5:** Statistical evidence supporting the pathway-level synthesis per study.

Study	Main Analysis Conducted	Main Statistical Values Related to Eligible Main Outcomes	Main Findings and Study Conclusions
(Bray et al., 2001)	Hierarchical regression of self-efficacy subtypes and fitness instructor efficacy predicting subsequent class attendance, stratified by exercise experience.	Exercise initiates: model R2 = 0.34, F(4,28) = 3.58, *p* = 0.02; step-2 R2 change for fitness instructor efficacy = 0.12, *p* = 0.03; barrier self-efficacy beta = 0.40, t = 2.40; fitness instructor efficacy beta = 0.38, t = 2.26. Experienced exercisers: model R2 = 0.01, F(4,89) = 0.27, *p* = 0.90.	For beginning exercisers, greater confidence in overcoming barriers and in instructor capability was associated with better attendance; for experienced exercisers, the psychosocial predictors did not explain attendance.
(Chermette et al., 2020)	Bivariate correlations and moderated regression of cohesion dimensions predicting seven-week attendance, with duration of membership as moderator.	Bivariate correlations with adherence were small and non-significant (ATG-S r = 0.04; ATG-T r = 0.09; GI-S r = 0.03; GI-T r = −0.03). Final interaction model: ATG-S × duration of membership beta = 0.14, t = 2.38, *p* = 0.018; F(1,271) = 5.67; R2 = 0.02.	Overall cohesion was not directly associated with attendance, but social attraction to the group was positively associated with attendance among members with longer group membership.
(Cheung et al., 2016)	Baseline self-efficacy predicting intervention-period class attendance and follow-up continuation of yoga practice.	Self-efficacy for Exercise with class attendance: rs = 0.34, *p* = 0.03. Self-efficacy for Exercise with follow-up home-practice days: rs = 0.06, *p* > 0.05. Self-efficacy for Exercise with follow-up home-practice minutes: rs = 0.01, *p* > 0.05.	Greater self-efficacy was associated with better class attendance during the eight-week yoga intervention, but not with continued follow-up practice after the group classes ended.
(Courneya & McAuley, 1995)	Direct social support and cohesion correlations with later attendance, plus indirect pathways tested in a trimmed path model.	Direct correlations with attendance were non-significant: social support composite r = 0.07; cohesion composite r = −0.17; subscales ranged from r = −0.17 to r = 0.11. Trimmed path model coefficients: social support to perceived behavioral control = 0.26; cohesion to attitude = 0.52; perceived behavioral control to intention = 0.32; attitude to intention = 0.31; intention to exercise adherence = 0.51.	Social support and cohesion were not directly associated with attendance, but the authors interpreted them as indirectly related to adherence through theory-of-planned-behavior mediators.
(Dyrlund & Wininger, 2006)	Mutually adjusted regression of class type and psychosocial variables predicting semester attendance.	Self-efficacy beta = 0.131, t = 1.92, *p* = 0.057; enjoyment beta = 0.018, t = 0.232, *p* = 0.817; relatedness beta = −0.242, t = −3.50, *p* = 0.001; overall model R2 = 0.286.	Relatedness showed an adjusted negative association with attendance, whereas self-efficacy and enjoyment were not significant.
(P. Estabrooks & Carron, 1998)	Hierarchical regression comparison of scheduling self-efficacy with perceived behavioral control and perceived barriers as predictors of later attendance.	Scheduling self-efficacy beta = 0.237; when entered at step 4 for weeks 9 through 12 attendance, R2 = 0.439 and F increment = 16.59, *p* < 0.01. Perceived behavioral control and perceived barriers did not add meaningful explanatory value.	Scheduling self-efficacy was positively associated with better later attendance and was a stronger protocol-relevant control-belief construct than perceived behavioral control or perceived barriers.
(P. A. Estabrooks & Carron, 1999)	Pearson correlations and stepwise regression of four cohesion dimensions predicting attendance at one, six, and twelve months. Team-building group-cohesion intervention compared with placebo and control conditions on attendance and post-hiatus return rate.	One month: attraction to the group—social r = 0.29, group integration—social r = 0.36, group integration—task r = 0.26, and group integration—social stepwise adjusted R2 = 0.12, F = 10.60. Six months: group integration—task r = 0.25 and stepwise adjusted R2 = 0.05, F = 4.76. Twelve months: group integration—task r = 0.25 and stepwise adjusted R2 = 0.05, F = 4.23. Attendance: control 65.0% (SD 32.1), placebo 70.0% (SD 20.5), team-building 90.8% (SD 13.8); overall F(2,30) = 4.10, *p* < 0.05; effect sizes for team-building versus control = 1.20 and versus placebo = 1.10. Return rate: control 40.0%, placebo 73.0%, team-building 91.7%; overall F(2,30) = 4.01, *p* < 0.05; team-building versus control effect size = 1.20.	Social and task cohesion were relevant to short-term attendance, but only group integration. Task remained associated with adherence over longer follow-up periods. The cohesion-focused team-building condition improved both short-term attendance and longer-term return to the program after a hiatus, although group-cohesion scale reliability was poor in this sample.
(P. Estabrooks & Carron, 1999)	Direct cohesion-attendance correlations and theory-of-planned-behavior regressions in older adult exercisers.	Direct attendance correlations: attractions to the group—social r = 0.09 and attractions to the group—task r = 0.05, both not statistically significant. Contextual hierarchical models: social cohesion to attitude R = 0.19, F(1,177) = 5.24, *p* < 0.05; task cohesion to perceived control R = 0.28, F(1,177) = 15.51, *p* < 0.01; intention and perceived control predicted attendance in final model F(2,176) = 19.23, *p* < 0.01.	Direct cohesion-attendance associations were not statistically significant.
(Feil et al., 2025)	Anticipated enjoyment predicting attendance at the next exercise class and maintenance over the next five classes, with contextual models adding intention.	Next-class attendance: OR = 1.40, 95% confidence interval 1.011 to 1.927, *p* = 0.022; after adding intention, OR = 1.06, *p* = 0.738 and intention OR = 2.05, *p* < 0.001. Maintenance: b = 0.301, *p* = 0.005; after adding intention, b = 0.125, *p* = 0.310 and intention b = 0.322, *p* < 0.001.	Anticipated enjoyment showed small positive direct associations with attendance and maintenance, but intention appeared to mediate these associations.
(Fraser & Spink, 2002)	Social support and group cohesion differentiating high versus low attenders and graduates versus dropouts in a clinical exercise program.	High versus low attendance function: Wilks lambda = 0.559, chi-square(3) = 13.65, *p* = 0.003; reliable alliance coefficient = 1.382, *p* = 0.009; attraction to the group—task coefficient = 0.826, *p* = 0.008; guidance coefficient = −1.207, *p* = 0.004. Dropout function: Wilks lambda = 0.829, chi-square(3) = 8.54, *p* = 0.04; reliable alliance coefficient = −1.027.	Reliable alliance consistently supported better compliance; task attraction supported better attendance, whereas higher guidance characterized poorer attendance.
(Hawley-Hague et al., 2014)	Multivariable prediction of attendance and adherence from participant, group-cohesion, and instructor characteristics.	Three-month attendance: ATG-S B = 0.321, *p* < 0.05; GI-T B = −0.343, *p* < 0.001; female instructor gender B = −4.396, *p* < 0.001; instructor age B = −0.139, *p* < 0.05; instructor experience B = 0.053, *p* < 0.05; motivational training B = 2.631, *p* < 0.05; extraversion B = −2.455, agreeableness B = −3.804, conscientiousness B = 1.746, intellect B = −0.554, all *p* < 0.05. Six-month attendance: ATG-S B = 0.699, *p* < 0.001; GI-T B = −0.494, *p* < 0.001; extraversion B = −1.381, *p* < 0.05; conscientiousness B = 2.811, *p* < 0.001. Six-month adherence: ATG-S OR = 1.87, *p* = 0.07; instructor experience OR = 0.96, *p* < 0.05; conscientiousness OR = 4.87, *p* < 0.05.	Both group cohesion and instructor characteristics mattered. Social attraction to the group predicted better attendance, whereas some task-bonding and several instructor personality or demographic characteristics predicted poorer attendance.
(Hewett et al., 2019)	Baseline predictors of adherence to sixteen weeks of Bikram yoga within the intervention arm of a randomized trial.	Participants attended 27 ± 18 classes on average. Exercise self-efficacy regression coefficient = 0.16, r = 0.042, *p* = 0.829. Final multivariable model: age beta = 0.492, *p* = 0.006; heart rate variability total power beta = 0.413, *p* = 0.021; pain beta = 0.329, *p* = 0.048.	The exercise self-efficacy measure was not associated with adherence. The study highlighted older age, lower pain, and higher heart rate variability as adherence predictors, and reported lack of enjoyment as a qualitative barrier.
(Izumi et al., 2015)	Generalized Estimating Equation models of leader behaviors, task cohesion, and social cohesion predicting walking-group participation over repeated follow-up assessments.	Leader behaviors: beta = 2.71, standard error = 0.60, *p* < 0.001. Task cohesion: beta = 0.28, standard error = 0.58, *p* = 0.63. Social cohesion: beta = 1.53, standard error = 0.37, *p* < 0.001. In joint model with social cohesion, leader behaviors beta = 1.81, standard error = 0.76, *p* = 0.02; social cohesion beta = 1.05, standard error = 0.42, *p* = 0.01.	Participants reporting more favorable Community Health Promoter behaviors and greater social cohesion had more consistent walking-group participation; social cohesion partially mediated the leader-behavior relationship, whereas task cohesion did not predict participation.
(Loughead et al., 2001)	Regression and correlation models of class leader behaviors and cohesion subscales predicting attendance over the prior four weeks.	Leader enthusiasm beta = 0.26, t(1,109) = 2.85, *p* < 0.01. Leader motivation beta = 0.29, t(1,109) = 3.16, *p* < 0.01. Individual attractions to the group—task r = 0.284, *p* < 0.01. Group integration—task r = 0.277, *p* < 0.01. Group integration—social r = 0.279, *p* < 0.01.	More favorable leader enthusiasm and motivation and stronger task and social integration were associated with better attendance. Task-related cohesion partially mediated some associations between leader enthusiasm and attendance.
(McAuley et al., 2011)	Structural equation model testing executive function and self-regulatory strategy use as antecedents of exercise-specific self-efficacy and long-term attendance.	Self-efficacy to attendance: beta = 0.34, z = 6.31, *p* < 0.01. Self-regulatory practices to self-efficacy: beta = 0.21, z = 4.33, *p* = 0.015. Dual-task error to self-efficacy: beta = −0.25, z = −4.54, *p* = 0.01. Stroop interference to self-efficacy: beta = 0.18, z = 4.04, *p* = 0.02. Model fit: chi-square = 141.40 (103), CFI = 0.96, RMSEA = 0.05, SRMR = 0.06.	Higher exercise-specific self-efficacy was associated with better attendance across the final eleven months of the program. Executive function and self-regulatory strategy use influenced adherence indirectly through self-efficacy.
(Shields et al., 2006)	Hierarchical mediation and additive regression models testing scheduling self-efficacy in relation to attendance across early and late program periods.	Early self-efficacy to early attendance: adjusted R-squared = 0.02, *p* = 0.079; overall additive model adjusted R-squared = 0.04, *p* = 0.019. Late self-efficacy to late attendance: adjusted R-squared = 0.09, *p* = 0.001; full mediation model adjusted R-squared = 0.10, *p* = 0.006.	Scheduling self-efficacy showed a weak, non-significant association with early attendance but a significant positive association with later attendance, and it fully mediated the relationship between early causal attributions and later attendance.
(Speed-Andrews et al., 2012)	Baseline self-efficacy contrast predicting yoga attendance proportion across the program.	Self-efficacy score of 7 versus 6 or lower: 88.3% versus 60.3% attendance; mean difference 28.0 percentage points; 95% confidence interval 10.6 to 45.4; *p* = 0.003.	Greater self-efficacy was associated with better Iyengar yoga adherence in breast cancer survivors.
(Steffens et al., 2019)	Prospective association of instructor identity leadership and exercise-group identification with later class attendance.	Group identification to attendance: b = 0.69, standard error = 0.23, beta = 0.23, t = 3.06, *p* < 0.01. Identity leadership indirect effect to attendance through group identification: gamma = 0.32, standard error = 0.11, 95% confidence interval 0.14 to 0.57.	Identity leadership was related to more frequent class attendance indirectly through stronger group identification.
(Watson et al., 2004)	Pre-intervention versus during-intervention attendance in a cohesion-focused team-building program.	Attendance increased from 53.5% to 75.7%; absolute increase 22.2 percentage points; t(11) = −2.15; *p* < 0.10.	Attendance improved during the team-building period, but the result did not meet the conventional 0.05 significance threshold.
(Wininger, 2004)	Multivariable prediction of step-aerobics attendance from self-efficacy and enjoyment.	Self-efficacy: beta = 0.383, t = 3.272, *p* = 0.002. Retrospective enjoyment: beta = 0.076, t = 0.673, *p* = 0.503. Overall model explained 20% of the variance in attendance.	Higher self-efficacy predicted more frequent attendance, whereas retrospective enjoyment did not.
(Yeung & Hemsley, 1997)	Stepwise regression of eight-week aerobics attendance on personality and efficacy cognitions.	Exercise-specific self-efficacy: estimate = −0.47, standard error = 0.13, t = −3.57, *p* = 0.001; the scale was negatively scored. Extraversion also predicted poorer attendance. Model R-squared = 0.312.	Greater exercise-specific self-efficacy was associated with better attendance after harmonizing the negatively scored efficacy measure.

**Table 6:** Comparative non-pooled synthesis of direction, magnitude, and robustness across behavioral pathways.

Behavioral Pathway	Studies (n)	Directional Consistency	Magnitude from Reported Estimates	Interpretation
Cognitive/action-control pathway: self-efficacy and perceived capability	10 studies	Most consistent positive pattern across domains, but not uniformly positive	Reported positive estimates included rs = 0.34; β/path coefficients around 0.24–0.40; adjusted or model-based R^2^ contributions around 0.02–0.09 in some models; one dichotomized contrast showed a 28-percentage-point attendance difference. Near-null effects were also reported, including r = 0.042 in one yoga intervention study.	Most robust pathway overall. The signal was strongest when self-efficacy was behavior-specific and temporally aligned with class attendance. Robustness was reduced by heterogeneity in efficacy constructs, outcome windows, adjustment strategies, and non-comparable effect measures.
Social-relational and instructor-shaped pathway: cohesion, groupness, social support, relatedness, group identification, instructor behavior	Groupness/cohesion: 11 studies; social support/relatedness: 3 studies; instructor factors: 5 studies	Mixed but suggestive; more favorable for specific social attachment and instructor/identity constructs than for broad social climate measures	Broad cohesion and social-support correlations were often near-null or small. More specific constructs showed stronger patterns, including social attraction/group integration correlations around r = 0.25–0.36, group identification β = 0.23, and leader-behavior coefficients that remained significant in some multivariable models. One team-building study reported large effects, but with serious risk-of-bias concerns.	Moderately supportive but context-dependent. Effects appeared strongest for social attraction, reliable alliance, social cohesion, group identification, and instructor/leader behavior. Generic cohesion or social support measures were less reliable.
Affective-motivational pathway: enjoyment and affective response	3 studies	Sparse and inconsistent	Anticipated enjoyment showed a small positive association with next-class attendance, but the effect attenuated after adding intention. Broader or retrospective enjoyment estimates were near-null in available adjusted models.	Weakest independent evidence. The pathway may be under-measured rather than unimportant, because available studies rarely used event-proximal or repeated affective assessment linked to specific attendance decisions.

## Data Availability

All data is presented in the article, since no original data was created.

## Abbreviations

The following abbreviations are used in this manuscript:

PRISMA	Preferred Reporting Items for Systematic Reviews and Meta-Analyses
ROBINS-I	Risk Of Bias In Non-randomised Studies—of Interventions
JBI	Joanna Briggs Institute
